# *Periplanta americana* extract regulates the Th17/Treg cell balance via Notch1 in ulcerative colitis

**DOI:** 10.3389/fphar.2024.1534772

**Published:** 2025-01-22

**Authors:** Yanqiu Zheng, Huibiao Li, Shiyu Qi, Fan Xiao, Jinbin Song, Shiyin Liu, Xinlin Chen, Yanwu Li, Muyuan Chen

**Affiliations:** ^1^ The First Affiliated Hospital, Guangzhou University of Chinese Medicine, Guangzhou, China; ^2^ Science and Technology Innovation Center, Guangzhou University of Chinese Medicine, Guangzhou, China; ^3^ School of Basic Medical Sciences, Guangzhou University of Chinese Medicine, Guangzhou, China

**Keywords:** ulcerative colitis, *Periplaneta americana* extract, Th17/Treg, Notch1, colonic mucosa

## Abstract

**Background:**

*Periplanta americana* extract (PAE), a traditional Chinese medicine (TCM) from *Shen Nong Ben Cao Jing*, has been used to treat ulcerative colitis (UC), various types of wounds and ulcers, infantile malnutrition, palpitation, asthma, and so on. However, the exact mechanisms of PAE in UC have still not been fully revealed. The study aims to explore the therapeutic effects and mechanisms of PAE in UC.

**Methods:**

The efficacy of PAE was evaluated using a DSS-induced UC mice model and the colon inflammation and mucosal barrier were comprehensively assessed. Furthermore, Network pharmacological analysis was utilized to identify potential targets and signaling pathways of PAE in the UC treatment. The proportion and the markers of Th17 and Treg cells in the spleen and colon were examined. The signal transduction was detected *in vivo*. *In vitro*, an activated Notch1-mediated Th17/Treg was modeled, and the effect of PAE on the epithelial cell barrier was examined.

**Results:**

PAE mitigated colon inflammation and intestinal barrier damage in UC mice. Network pharmacological analysis showed that the targets of UC intervention by PAE may be closely related to Th17 cell differentiation, the IL-17 signaling pathway, and cytokine-cytokine receptor interaction. Mechanistically, PAE regulated the balance of Th17/Treg and inhibited the Notch1/Math1 pathway in the colon of UC mice. *In vitro*, PAE intervention alleviated the activated Notch1-mediated Th17/Treg imbalance in Jurkat T cells. After notch1-activated Jurkat T cells were co-cultured with HCoEpic cells, the expressions of Occludin, ZO1 were higher in the HCoEpic cells.

**Conclusion:**

PAE could alleviate colon inflammation and mucosal barrier damage in UC, which are related to the inhibition of Notch1 and the regulation of the Th17/Treg balance. PAE might be a potential candidate agent for UC treatment.

## 1 Introduction

Ulcerative colitis (UC) is an idiopathic, chronic inflammatory disorder of the colonic mucosa, whose etiology is related to the interaction between the immune system, environmental factors, dietary factors, and genetics resulting in mucosal inflammation (Ordás et al., 2012; Fritsch et al., 2021). UC affects 5 million people worldwide and the incidence is increasing (Le Berre et al., 2023). Medical management goals are first, to induce rapid clinical response and normalize biomarkers, and second, to maintain the clinical remission. Treatments for inducing remission include 5-aminosalicylic acid drugs and corticosteroids. Maintenance treatments include 5-aminosalicylic acid drugs, thiopurines, biologics (anti-cytokines and anti-integrins), and small molecules (Janus kinase inhibitors and sphingosine-1-phosphate receptor modulators) (Raine et al., 2022; Feuerstein et al., 2020). However, remission rates do not surpass 20%–30% in induction clinical trials and 30%–60% of patients in a real-life setting, despite expanding treatment options (Le Berre et al., 2023). Therefore, it is imperative to develop novel and alternative therapeutic agents for treating UC.

The Th17/Treg balance played an important role in the development of autoimmune diseases by regulating the immune responses, and it was involved in the pathological changes of local inflammation in the body (Lee, 2018). The clinical observation study suggested that the inflammation level was positively correlated with the level of IL-17 and Th17, which was negative correlation with Treg and TGF-β1 (Gong et al., 2016). Th17/Treg-mediated immunological responses in the intestinal microenvironment were one of the causes of UC, and it has also become a hot spot in studying the pathogenesis of UC in recent years (Yan et al., 2020). In human and experimental UC, recovering the Th17/Treg immune balance may contributed to disease remission in UC (Huang et al., 2022; Lv et al., 2023). Furthermore, the Notch signaling pathway was highly conserved in multicellular organisms, which can regulate T cell development and differentiation. Notch signal activation can affect Th17/Treg immune imbalance (Yang et al., 2023).

*Periplaneta americana* (PA)/*Periplaneta americana* extract (PAE), a traditional Chinese medicine (TCM) of animals, has been recorded in *Shen Nong Ben Cao Jing* (25–220 A.D.). It can be used to treat various types of wounds and ulcers, infantile malnutrition, palpitation, asthma, and so on (Feng et al., 2023). Of note, the mucosal restorative effects of PAE have attracted significant attention. In a previous experiment, PAE exhibited good anti-UC activity in mice (Xie et al., 2023). However, the exact mechanisms of PAE on UC are still not fully revealed. In this study, we examined the therapeutic effect of PAE on UC and how PAE regulates Th17/Treg cell balance through the Notch1 signaling *in vivo* and *in vitro*. This study provides a new theoretical and experimental basis for the promotion and application of PAE.

## 2 Materials and methods

### 2.1 Preparation of insect material

The extraction method of PAE using ethanol was referred from a previous study (Song et al., 2017). In this study, PAE was provided by the Department of Pharmaceutical Preparation, the First Affiliated Hospital of Guangzhou University of Chinese Medicine. PA (Yunfeng *Periplaneta americana* breeding base, Yunnan, China, Lot: 20210730) dried powder was extracted with 85% ethanol three times at 60°C. After standing overnight at 4°C, the upper layer of oil was removed. Petroleum ether was added to extract the active ingredient of PAE.

### 2.2 Animals and treatment

BALB/c male mice (16–20 g, 6–8 weeks old) were obtained from Guangdong Medical Laboratory Animal Center (License number: SCXK, Guangdong, 2022-0002). These animals were housed in the specific pathogen-free (SPF) animal laboratory of Guangzhou University of Chinese Medicine (License number: SYXK, Guangdong, 2018-0001). In this study, all protocols for animal experiments were approved by the ethics committee of Guangzhou University of Chinese Medicine (20220526013).

**FIGURE 1:**
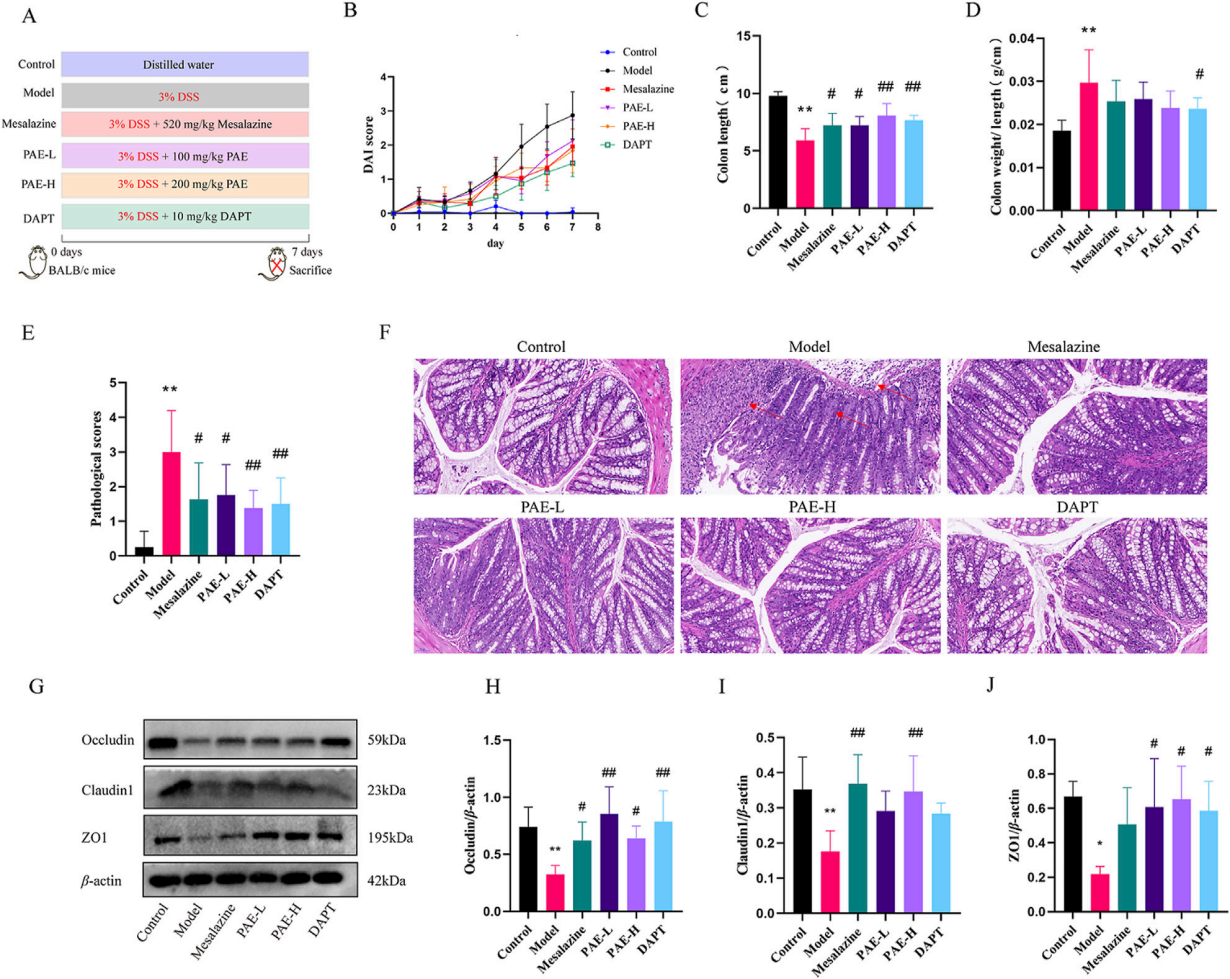
PAE mitigates the symptoms and intestinal barrier damage in UC mice. **(A)** The schematic design of the experiment. **(B)** Disease activity index. **(C)** The colon length. **(D)** The colon weight/length. **(E)** Colon pathological scores. **(F)** Histopathology alterations in the colon (HE, ×200). Red arrows indicate the destruction of colon mucosal. **(G)** Western blot images of occluding, claudin1, and ZO1 in colon. **(H–J)** Analysis of gray-scale of occluding, claudin1, and ZO1 protein expression from all groups. (n = 4–8, *P < 0.05 and **P < 0.01 versus control group, ^#^ *P* < 0.05 and ^##^ *P* < 0.01 versus model group).

Mice were randomly assigned to six groups (Figure 1A): control, model, mesalazine, low-dose of PAE (PAE-L), high-dose of PAE (PAE-H), and DAPT (a Notch1 inhibitor) group. The UC model was established using 3% dextran sulfate sodium (DSS, MP Biomedicals, lot number: 160110) for 7 days. Mesalazine (520 mg/kg/day), PAE-L (100 mg/kg/day), PAE-H (200 mg/kg/day), and DAPT (10 mg/kg/day) were respectively administered via the intragastric route for 7 days.

In this experiment, the method of euthanasia of experimental animals is based on national standards of the Laboratory animal—Guidelines for euthanasia (GB/T 39760-2021). Mice were anesthetized by intraperitoneal injection of sodium pentobarbital once time (150 mg/kg). When the mouse’s heart stops beating, the colon and spleen tissue was collected for further study.

### 2.3 Disease activity index

Disease activity index (DAI) = (weight loss percentage score + stool score + rectal bleed score)/3 (Guo et al., 2022). The occult blood test paper (Baso, Zhuhai, China, Lot: BA2020B) was assessed based on the timing of positive results using the Pyramidone Chemical Method (Supplementary Table S1).

### 2.4 Histopathological evaluation

The middle part of the colon (1 cm) was fixed in 4% paraformaldehyde solution for 24 h, embedded in paraffin, and cut into sections no larger than 0.3 mm thick. Hematoxylin and eosin (HE) staining was performed, and then pathological scores were determined by the severity of colonic mucosal injury on a scale of 0–4 according to Yan et al.’s report (Yan et al., 2022). The scoring table is shown in Supplementary Table S2.

### 2.5 Network pharmacology

Currently, due to the close relationship of basic principles, network pharmacology has been widely applied in deciphering the physiological activity mechanisms of TCM (Wang et al., 2021). We further predicted the targets and pathways for PAE intervention in UC using network pharmacological analysis. The chemical constituents of PA were searched by the Traditional Chinese Medicine Systems Pharmacology (TCMSP) database (http://tcmspw.com/tcmsp.php), Pubmed database (https://pubmed.ncbi.nlm.nih.gov/) and CNKI database (https://www.cnki.net/) using the keywords of “*Periplaneta americana*”, “Kangfuxinye” which is a patent medicine with PAE as its main component. Both the target of PAE chemical constituents and UC were obtained by the UniProt database (https://www.uniprot.org/) or CTD (Comparative Toxicogenomics Database, https://ctdbase.org/). The targets of PAE and UC were combined using the VENNY2.1 analysis tool (https://bioinfogp.cnb.csic.es/tools/venny/). The protein-interaction data of PAE-related targets and UC were obtained from the STRING database https://string-db.org/). In addition to the above, Cytoscape (https://cytoscape.org/) was used to build the Protein-Protein Interaction (PPI) and analyze the metabolic pathways of the related targets of PAE in the treatment of UC. Furthermore, Gene Ontology (GO) and Kyoto Encyclopedia of Genes and Genomes (KEGG) were used to analyze the metabolic pathways of the related targets of PAE in the treatment of UC by DAVID database (https://david.ncifcrf.gov/tools.jsp).

### 2.6 Flow cytometry assay

The spleen was prepared as single-cell suspensions, then stimulated and blocked with PMA (Lot: CS1001) and BFA (Lot: CS1002), which were purchased from MultiSciences (Lianke) Biotech, Hangzhou, China. The following antibodies were used for surface staining for 30 min at 4°C: FITC anti-CD4 (BioLegnd, San Diego, CA, United States, Lot: 100509), PE/Cyanine7 anti-CD25 (BioLegend, Lot: 102016). For intracellular staining with Alexa Fluor 647 anti-Foxp3 (BioLegend, Lot: 126408) and PE anti-IL-17A (BioLegend, Lot: 506904), cells were fixed and permeabilized with a True-Nuclear™ Transcription Factor Buffer Set (BioLegend, Lot: 424401). Samples were acquired and analyzed with a CytoFLEX LX flow cytometer (Beckman Coulter, California, United States) and the data was analyzed with CytExpert software. CD4^+^ CD25^+^ Foxp3^+^ cells as Treg cells were evaluated and CD4^+^ IL-17A^+^ cells were labeled as Th17 cells according to previous studies (Yu et al., 2015).

### 2.7 Western blotting

Tissues or cells were lysed with protein lysis buffer (KeyGEN BioTECH, KGP2100) containing proteinase and phosphatase inhibitors, and subsequently, the concentration of protein was quantified with BCA assay (KeyGEN BioTECH, KGP902). The equal amount of protein was separated with sodium dodecyl sulfate -polyacrylamide gel electrophoresis and transferred to polyvinylidene fluoride membranes (Merck Millipore, LG18-1501M-1H). After blocking with 5% skim milk for 1.5 h (h) at room temperature, the membranes were incubated overnight at 4°C with different primary antibodies against Occludin (13409-1-AP, Proteintech), Claudin 1 (13050-1-A, Proteintech), ZO1 (TA5145F, Abmart; 21773-1-A, Proteintech), Foxp3 (Ab215206, Abcam), RORγt (13205-1-AP, Proteintech), Math1 (21215-1-A, Proteintech), Notch1 (T55244S, Abmart; 20687-1-AP, Proteintech), Hes1(11988S, CST) and β-actin (ab179467; Abcam). Followed by secondary antibodies for 1 h. The PVDF membranes were detected using a chemiluminescence imager (Bio-Rad) and quantified using the Image Lab software (Version 4.1, Bio-Rad).

### 2.8 Immunofluorescence or immunohistochemistry

As described in immunohistochemistry (IHC) immunofluorescence (IF) by Xiao et al. (2024), the blank sections were dewaxed, antigen retrieval in turn. After blocking with 5% bovine serum albumin (BSA), the primary antibody was incubated at 4°C overnight, and the secondary antibody was incubated for 30 min at 37°C. Finally, diaminobenzidine (DAB) was used to stained IHC samples, or IF with DAPI.

### 2.9 Cell viability assay and modeling

Jurkat cells (Pricella, CL-0129) were cultured in RPMI-1640 medium with 10% fetal bovine serum (FBS) at 5% CO_2_, 37°C (Yang et al., 2022). The cells were treated with the Notch1 agonist valproic acid (VPA. Selleck, S1168) in different concentrations (0.4, 0.8, 1.6, 3.2, 6.4, 12.8 mM) for 24, 48 h in 96 wells-plates. Next, CCK-8 reagent (GLPBIO, GK10001) was added into each well for 4 h. The optical density values were measured at 450 nm using a microplate reader. For more details about PAE (0.01, 0.1, 1, 10 mg/mL) processing, refer above.

Jurkat T cells were treated with PBS, VPA (3.2 mM VPA), and PAE (3.2 mM VPA+1 mg/mL PAE) for 24 h. Then notch1-activated Jurkat T cells were co-cultured with HCoEpic cells for 10 h.

### 2.10 Statistical analysis

The results are the mean ± standard deviation. Data were analyzed by SPSS 19.0 (IBM, Armonk, NY, United States). One-way analysis of variance was used for the comparison of data between groups. *P* < 0.05 was considered statistically significant.

## 3 Results

### 3.1 PAE mitigates the symptoms and intestinal barrier damage in UC mice

Compared with the control group, the DAI score of the model group was significantly higher on the second day with 3% DSS treatment. Compared with the model group, lower DAI scores were observed in the mesalazine, PAE-L, PAE-H, and DAPT groups (Figure 1B). As illustrated in Figures 1C, D, the colon of the model group was obviously wrinkled, and the unit weight of colon in the model group (colon weight/length) was increased compared with the control group (*P* < 0.01). Treatment with mesalazine, PAE-L, PAE-H and DAPT significantly increased the colon length in UC mice (*P* < 0.05, *P* < 0.01). In addition, there was a significant decrease in the unit weight of the DAPT group compared to the model group (*P* < 0.05), while there was no statistical significance in the mesalazine and PAE groups (*P* > 0.05).

HE Staining showed intestinal barrier damage in the DSS-included UC mice, including disordered mucosal gland structure and diminished glands and goblet cells. In addition, a large number of inflammatory cells infiltrated in the mucosal and submucosal layers, individually involving the muscular layer. Damage to the colon intestinal mucosa was improved with the treatment of PAE-L, PAE-H, and DAPT (Figures 1E, F).

Tight junctions (TJs) are essential for the formation and maintenance of the intestinal barrier by enhancing epithelial cell-cell adhesion (Balda and Matter, 2023). The levels of tight junction proteins, including occludin, claudin1, and ZO1 were further measured to evaluate the injury of colonic mucosa in UC mice. It was found different degrees of upregulation of occludin, claudin1, and ZO1 levels in UC mice with PAE, mesalazine, and DAPT treatment (*P* < 0.05, *P* < 0.01), suggesting that PAE and DAPT could protect the colonic mucosal barrier by increasing the expression of occludin, claudin1, and ZO1 (Figures 1G–J).

### 3.2 Pathway and function analyses for potential targets of PA acting on UC

**FIGURE 2:**
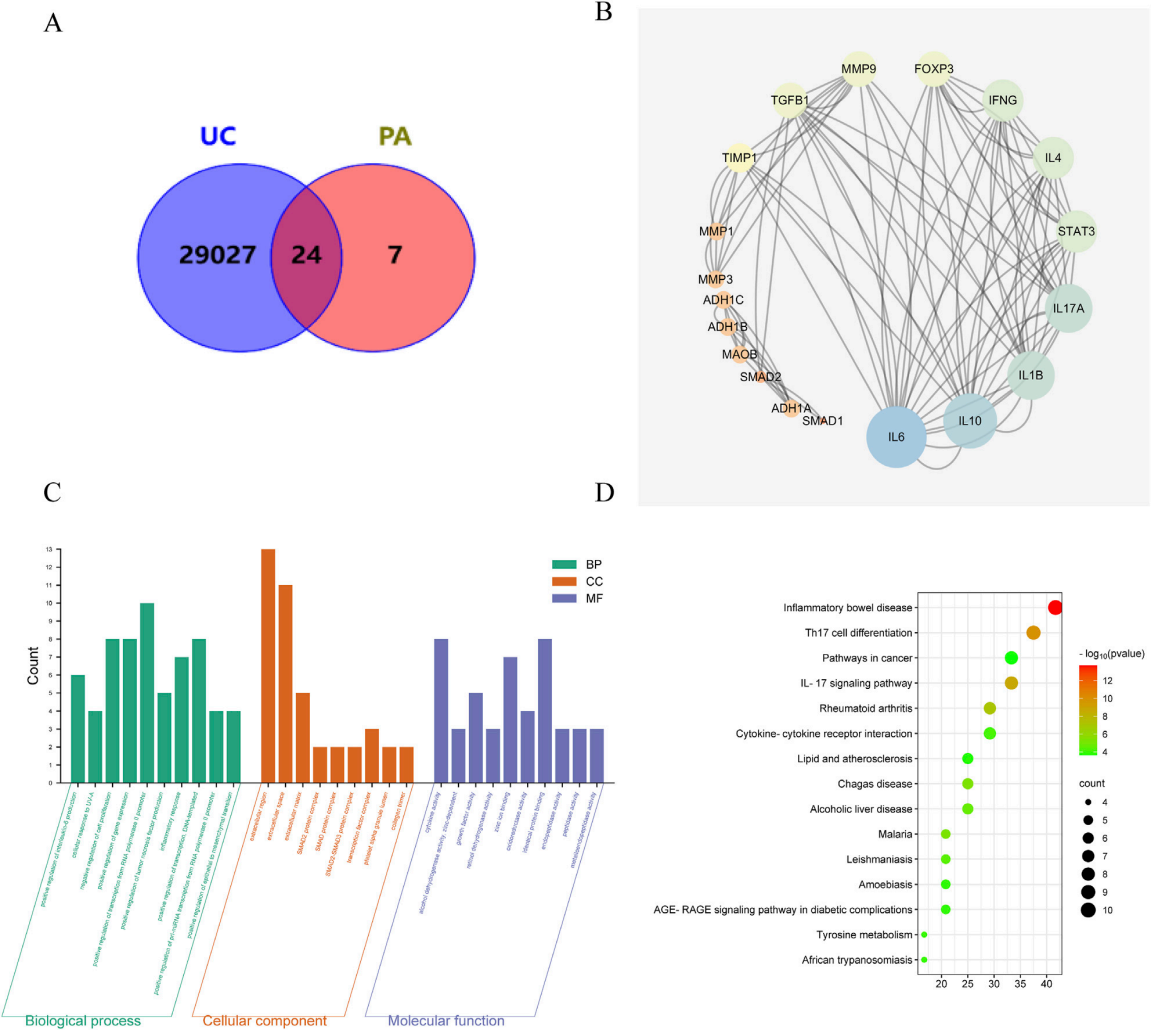
Pathway and function analyses for potential targets of PA acting on UC. **(A)** Venn diagram of targets for PA acting on UC. **(B)** PPI network analysis. **(C)** GO enrichment. **(D)** KEGG pathway enrichment.

A total 4 components were identified in PAE by high-performance liquid chromatography (HPLC), including *uracil* (C_4_H_4_N_2_O_2_), *hypoxanthine* (C_5_H_4_N_4_O), *xanthine* (C_5_H_4_N_4_O_2_) and *inosine* (C_10_H_12_N_4_O_5_) (Supplementary Figure S1; Supplementary Table S3). In addition, *cytosine* (C_4_H_5_N_3_O) and *cytidine* (C_9_H_13_N_3_O_5_) were supplemented through PubMed and CNKI databases. A total of 31 targets of PA were identified from the TCMSP database, and 29, 051 disease targets were yielded in the CTD database. After overlapping of common targets with Venn diagrams, 24 common targets of the active ingredient of PA targeting UC were obtained (Figure 2A). Furthermore, the PPI network showed the top ten targets including IL-6, IL-10, IL-1B, IL-17A, STAT3, IL-4, IFNG, FOXP3, MMP9, and TGFβ1 (Figure 2B).

GO enrichment identified that UC intervention by PA may be related to the positive regulation of IL-6 production, negative regulation of cell proliferation, and factor activity (Figure 2C). KEGG enrichment analysis showed that the targets of UC intervention by PA may be closely related to Th17 cell differentiation, the IL-17 signaling pathway, and cytokine-cytokine receptor interaction (Figure 2D).

### 3.3 PAE regulates the balance of Th17/Treg cells in UC mice

**FIGURE 3:**
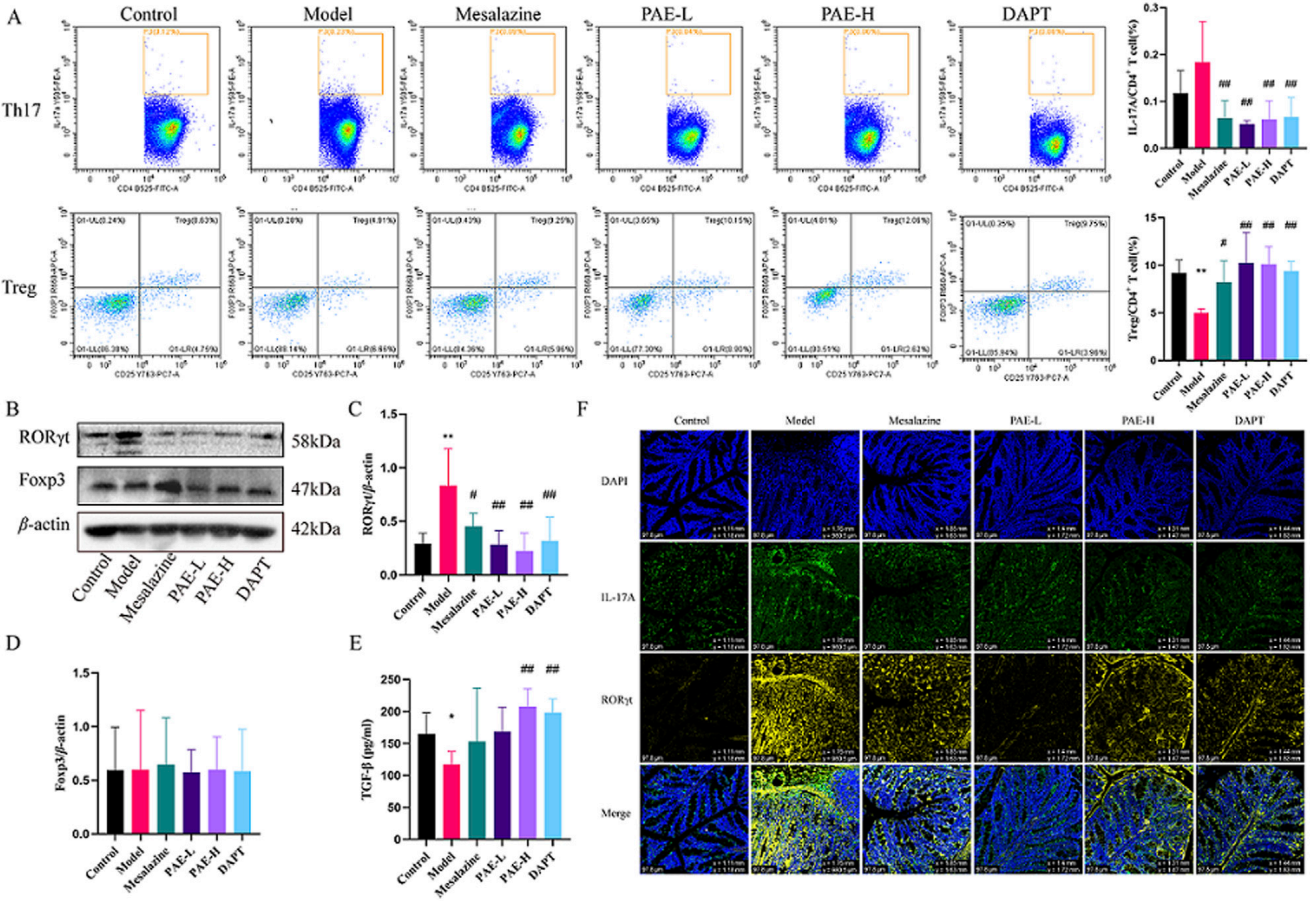
PAE regulates the balance of Th17/Treg cells in UC mice. **(A)** Flow cytometry analysis of Th17 (CD4^+^ IL-17A^+^) and Treg (CD4^+^ CD25^+^ Foxp3^+^) cells in spleen. **(B)** Western blot images of RORγt and Foxp3 in the colon. **(C, D)** Analysis of gray-scale of RORγt and Foxp3 protein expression. **(E)** Levels of TGF-β in peripheral blood. **(F)** Immunofluorescence images of RORγt and IL-17A in colon (×100). (n = 4–6, *P < 0.05 and **P < 0.01 versus control group, ^#^ *P* < 0.05 and ^##^ *P* < 0.01 versus model group).

The percentages of Th17 and Treg cells were detected by flow cytometry assay. The results showed that the percentage of Th17 cells was increased, while the percentage of Treg cells was remarkably decreased in the model group compared with the control group. After treatment with PAE, or DAPT, the percentage of Th17 cells was obviously reduced, and the percentage of Treg was increased compared with the model groups (*P* < 0.01) (Figure 3A).

RORγt and Foxp3 are the critical transcription factors for the development and function of Th17 and Treg cells, respectively. Furthermore, IL-17A and TGF-β are crucial Th17 and Treg cell effectors. As shown in Figures 3B–F, RORγt and IL-17A were significantly increased in the colon of the model group compared to the control (*P* < 0.05), while mesalazine, PAE-L, PAE-H and DAPT treatment dramatically decreased the expression of RORγt and IL-17A (*P* < 0.05, *P* < 0.01). TGF-β downregulated the expressions in the model group compared to the control (*P* < 0.05), while PAE and DAPT intervention could upregulated the expressions (*P* < 0.01). In addition, there was no statistical difference in Foxp3 expression between the groups (*P* > 0.05).

### 3.4 PAE inhibits the Notch1/Math1 pathway in the colon of UC mice

**FIGURE 4:**
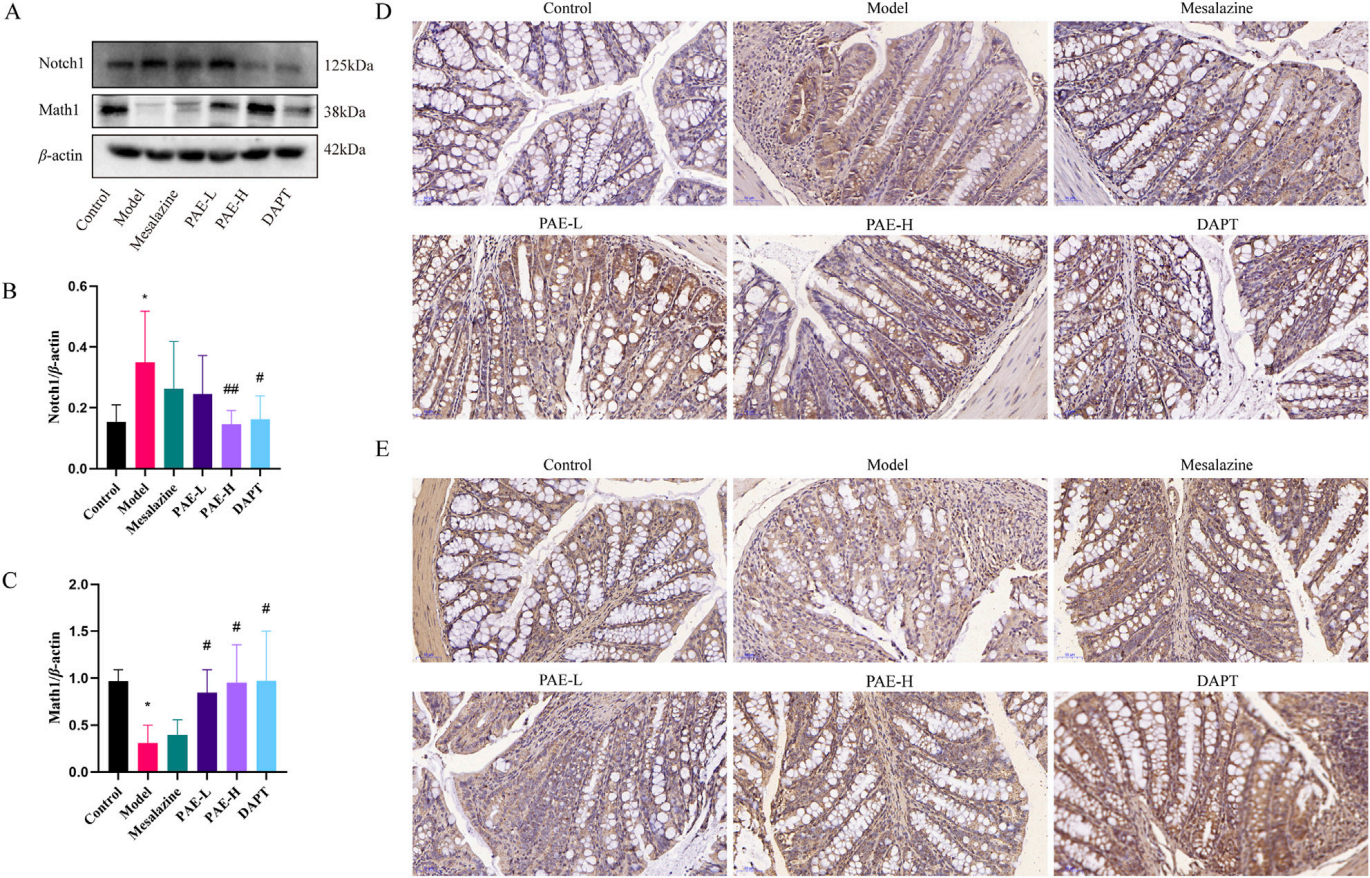
PAE inhibits the Notch1/Math1 pathway in UC mice. **(A)** Western blot images of Notch1 and Math1 in the colon. **(B, C)** Analysis of gray-scale of Notch1 and Math1 protein expression. **(D)** Immunohistochemistry images of Notch1 in colon (×100). **(E)** Immunohistochemistry images of Math1 in the colon (×100). (n = 3–6, *P < 0.05 and **P < 0.01 versus control group, ^#^ *P* < 0.05 and ^##^ *P* < 0.01 versus model group).

As seen in Figure 4, the protein expression of Notch1 was increased, and the Math1 was reduced in the colon of the model group compared with the control group. Conversely, Notch1 expression was downregulated and Math1 expression was upregulated in the PAE-H and DAPT groups compared with the model group (*P* < 0.05). It suggested that PAE could regulate DSS-induced Notch signaling activation in UC mice.

### 3.5 PAE attenuates the disruption of TJs in HCoEpic cells by regulating Notch1-Th17/Treg cells *in vitro*

**FIGURE 5:**
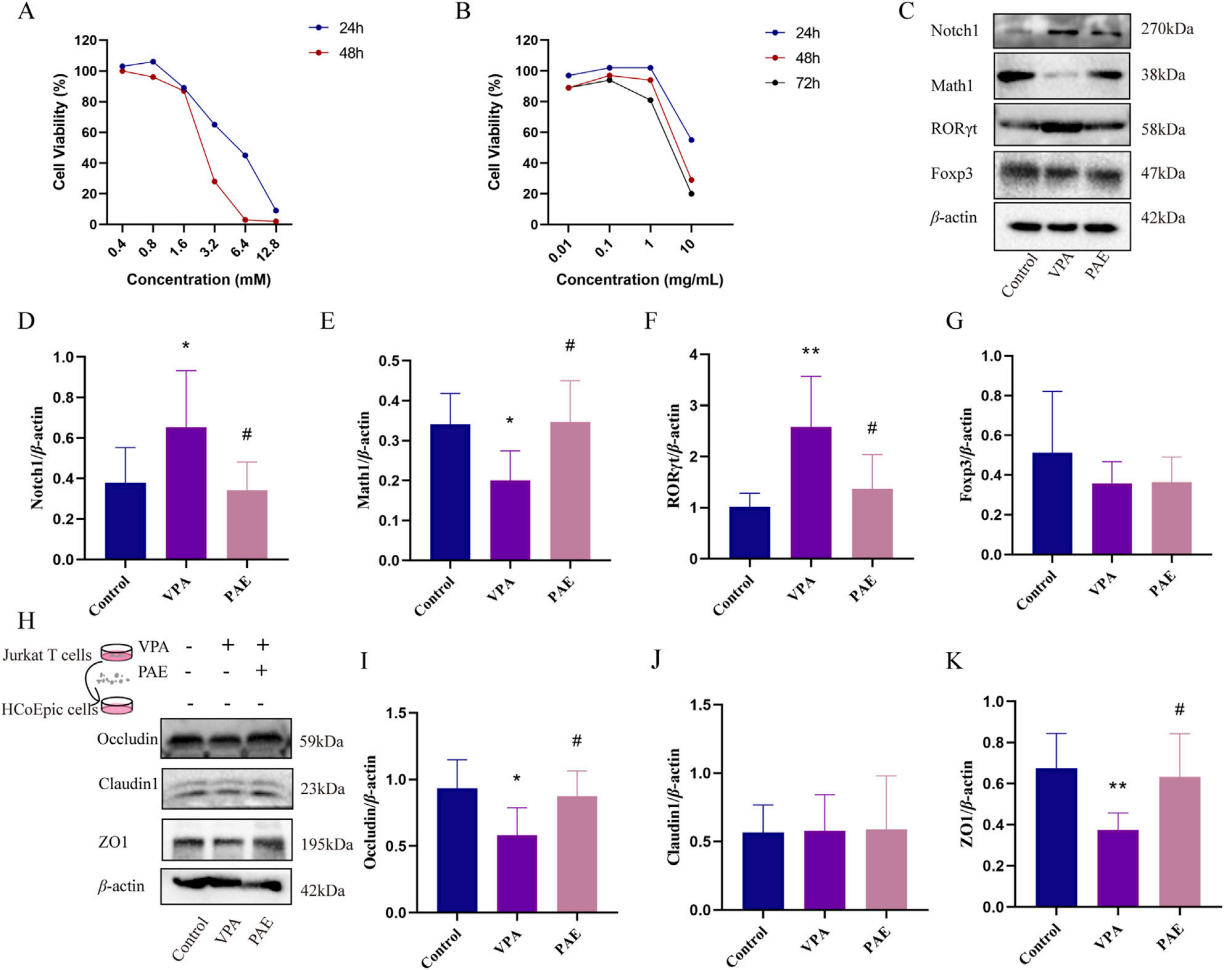
PAE attenuates the disruption of tight junction proteins in HCoEpic cells by regulating activated Notch1-mediated Th17/Treg imbalance *in vitro*. **(A)** Effect of VPA on the viability of Jurkat T cells analyzed with CCK8 assay. **(B)** Effect of PAE on the viability of Jurkat cells analyzed with CCK-8 assay. **(C)** Western blot images of Notch1, Math1, RORγt, and Foxp3 in Jurkat T cells. **(D–G)** Analysis of gray-scale of Notch1, Math1, RORγt, and Foxp3 protein expression. **(H)** After notch1-activated Jurkat T cells were co-cultured with HCoEpic cells, Western blot images of occluding, claudin1, and ZO1 in HCoEpic cells. **(I–K)** Analysis of gray-scale of occluding, claudin1, and ZO1 protein expression. (n = 5–8, *P < 0.05 and **P < 0.01 versus control group, ^#^ *P* < 0.05 and ^##^ *P* < 0.01 versus VPA group).

To identify a possible effect of VPA (a Notch1 agonist) and PAE *in vitro*, the proliferation of Jurkat T cells was analyzed with CCK-8 assay. It indicated that cell viability was greater than 80% at 0.4–1.6 mM VPA and highest at 0.8 mM for 24 h (Figure 5A). PAE (0.1 mg/mL and 1 mg/mL) administration significantly promoted cell proliferation for 24 h. The overall cell viability of different concentrations of PAE at 24 h was higher than that at 48 and 72 h (Figure 5B). To ensure cell viability as much as possible, we built the model using VPA (0.8 mM) and PAE (1 mg/mL) for 24 h.

VPA obviously upregulated the expressions of Notch1 and downregulated Math1 (*P* < 0.05). Meanwhile, the ratio of RORγt/Foxp3 was upregulated in the VPA group (*P* < 0.05). PAE intervention could alleviate this situation compared with the VPA group (*P* < 0.05) (Figures 5C–G).

To further determine the relationship between Notch1 and Th17/Treg in the colonic mucosal damage, Notch1-activated Jurkat T cells were co-cultured with HCoEpic cells. The results showed that the expressions of Occludin, ZO1 was higher in the PAE group compared with VPAs (*P* < 0.05) (Figures 5H–K).

## 4 Discussion

UC, a lifelong inflammatory disease, is characterized by recurrent inflammation of the intestinal mucosa, usually starting in the rectum and continuing proximally along the colon (Kaplan and Ng, 2016; Ungaro et al., 2017). PAE has the potential to regulate the expression of inflammatory and immune factors associated with the body’s mucosa, thereby facilitating a repair effect on the mucosa (Zeng et al., 2019; Nguyen et al., 2020). PAE accelerates wound healing by activating the TGF-β/Smad signaling pathway, promoting epithelial regeneration and collagen deposition (Chen et al., 2019). In addition, it promotes mucosal repair and healing by inhibiting inflammation, and promoting cell proliferation and collagen synthesis (Li et al., 2016). A meta-analysis found that the rehabilitation new liquid prepared by PAE significantly improved the clinically effective rate of UC treatment and reduced the recurrence rate (Li et al., 2018).

DSS is a negatively charged polysaccharide with a highly variable molecular weight. The small molecule DSS penetrates the mucus layer and interacts directly with intestinal epithelial cells. Macromolecule DSS forms complexes with medium-chain-length fatty acids (MCFAs), which fuses with colonocyte membranes to trigger inflammatory signaling in the distal colon (Yang and Merlin, 2024). In our study, intestinal barrier damage was observed in the DSS-included UC mice, including shedding colonic epithelium, disordered mucosal gland structure, diminished glands and goblet cells, and inflammatory cell infiltration in the mucosal and submucosal layers. Damage to the colon intestinal mucosa was improved with the treatment of PAE and DAPT. In addition, we found different degrees of upregulation of occludin, claudin1, and ZO1 levels in DSS-included UC mice with PAE and DAPT treatment. These results clearly indicated PAE and DAPT (a Notch1 inhibitor) could repair the mechanical barrier of intestinal mucosa by suppressing inflammatory infiltration and increasing the TJs in UC mice.

Based on the therapeutic effect of PAE on UC, network pharmacological analysis was used to predict the potential targets and pathways. PPI network showed the top ten targets including IL-6, IL-10, IL-1B, IL-17A, STAT3, IL-4, IFNG, FOXP3, MMP9, and TGFβ1. GO enrichment identified UC intervention by PA may be related to the positive regulation of IL-6 production, negative regulation of cell proliferation, and factor activity. KEGG enrichment analysis showed that the targets of UC intervention by PA may be closely related to Th17 cell differentiation, IL-17 signaling pathway, and cytokine-cytokine receptor interaction. PAE administration significantly inhibited the proportion of Th17 cells in the spleen, as well as the expression of RORγt and the secretion of IL-17A in colon of UC mice. Meanwhile, PAE administration upregulated the secretion of TGF-β. However, there was no statistical difference in Foxp3 expression with PAE treatment. We speculated that PAE is more obvious in inhibiting the differentiation of Th17 cells in the effect of regulating Th17/Treg immune balance. The efficacy in intestinal inflammation alleviation and mucosal protection was similar to mesalazine.

Notch signaling plays an important role in the differentiation of T cells into all known effector subsets such as Th1, Th2, Th9, Th17, Th22, and Treg. In an experimental model of autoimmune uveitis, DAPT improved Th17/Treg balance in lymph nodes, spleen, and eye tissues by inhibiting the Notch pathway (Yin et al., 2020). Similarly, we found that the percentage of Th17 cells was obviously reduced and the percentage of Treg was increased in UC mice with DAPT treatment. To further explore the effect of Notch1 signaling activation on T cell differentiation and intestinal epithelial cell barrier function, the Notch1 agonist VPA firstly activated Jurkat T cells, and co-culture with colon epithelial cells HCoEpic *in vitro*. PAE inhibits VPA-induced upregulation of RORγt in Jurkat cells, which in turn affects Th17 differentiation in lymphocytes. These results further validated that regulation of the Notch1 may be an effective means of improving the Th17/Treg balance and treating UC. Notch1 signaling may be a target for PAE to regulate Th17/Treg balance. Indeed, the effect of Th17 and its effector IL-17A on inflammatory diseases is not just a negative effect, but excessive inhibition of Th17 signaling will maintain the Th17 cytokine program and the inflammation it causes in a negative feedback loop (Tan et al., 2022). The inhibition of the Notch1 may be beneficial for UC, but the dosage and timing of drug intervention still need further research.

In this study, we elucidated important findings on PAE-treated UC and explored the molecular mechanisms. However, certain limitations within this study require further investigation. First, it is important to acknowledge potential limitations and omissions in data acquisition and network pharmacological analysis. In addition, further investigations should take into account the complicated metabolic processes of PAE within the body. Further *in vivo* and *in vitro* studies of PAE are needed in the future.

## 5 Conclusion

**FIGURE 6:**
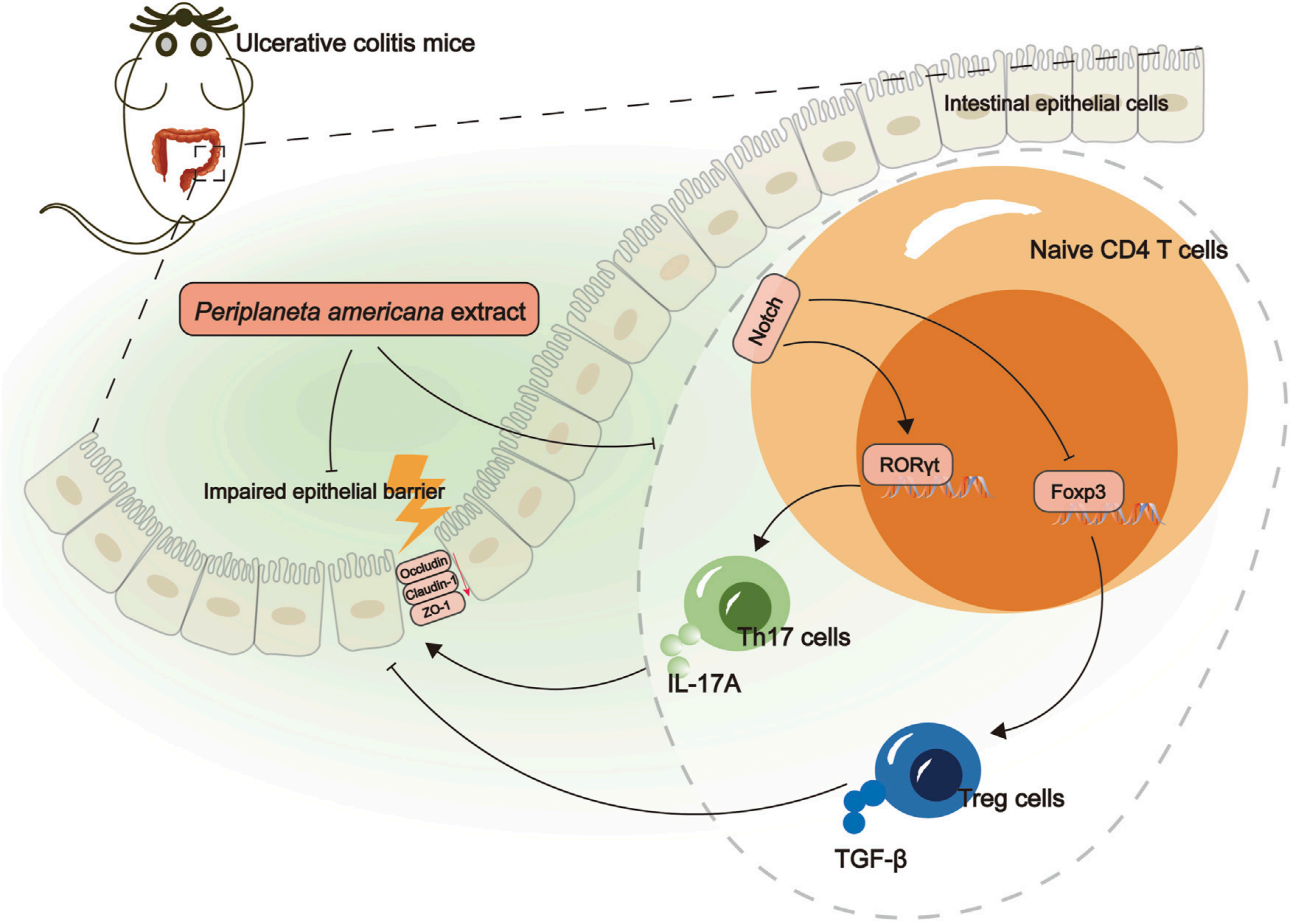
A Schematic Illustration of PAE alleviates DSS-induced ulcerative colitis.

The study demonstrated that PAE could alleviate colon inflammation and mucosal barrier damage in UC, which are related to the inhibition of Notch1 and the regulation of the Th17/Treg balance (Figure 6). Consequently, PAE might be a potential candidate agent for UC treatment.

## Data Availability

The original contributions presented in the study are included in the article/Supplementary Material, further inquiries can be directed to the corresponding authors.

## References

[B1] Balda M. S. Matter K. (2023). Tight junctions. Curr. Biol. 33, R1135–r1140. 10.1016/j.cub.2023.09.027 37935122

[B2] Chen Z. Hu Y. Li J. Zhang C. Gao F. Ma X. (2019). A feasible biocompatible hydrogel film embedding *Periplaneta americana* extract for acute wound healing. Int. J. Pharm. 571, 118707. 10.1016/j.ijpharm.2019.118707 31593807

[B3] Feng L. Yang H. Zhang S. Xu L. (2023). Research advances on the restorative effect of *Periplaneta americana* extracts on mucosa. Heliyon 9, e16623. 10.1016/j.heliyon.2023.e16623 37484248 PMC10360583

[B4] Feuerstein J. D. Isaacs K. L. Schneider Y. Siddique S. M. Falck-Ytter Y. Singh S. (2020). AGA clinical practice guidelines on the management of moderate to severe ulcerative colitis. Gastroenterology 158, 1450–1461. 10.1053/j.gastro.2020.01.006 31945371 PMC7175923

[B5] Fritsch J. Garces L. Quintero M. A. Pignac-Kobinger J. Santander A. M. Fernández I. (2021). Low-fat, high-fiber diet reduces markers of inflammation and dysbiosis and improves quality of life in patients with ulcerative colitis. Clin. Gastroenterol. Hepatol. 19, 1189–1199.e30. 10.1016/j.cgh.2020.05.026 32445952

[B6] Gong Y. Lin Y. Zhao N. He X. Lu A. Wei W. (2016). The Th17/Treg immune imbalance in ulcerative colitis disease in a Chinese han population. Mediat. Inflamm. 2016, 7089137. 10.1155/2016/7089137 PMC4763012 26977120

[B7] Guo H. Guo H. Xie Y. Chen Y. Lu C. Yang Z. (2022). Mo(3)Se(4) nanoparticle with ROS scavenging and multi-enzyme activity for the treatment of DSS-induced colitis in mice. Redox Biol. 56, 102441. 10.1016/j.redox.2022.102441 35985164 PMC9411672

[B8] Huang C. Mei Q. Lou L. Huang Z. Fu Y. Fan J. (2022). Ulcerative colitis in response to fecal microbiota transplantation via modulation of gut microbiota and Th17/Treg cell balance. Cells 11, 1851. 10.3390/cells11111851 35681546 PMC9180439

[B9] Kaplan G. G. Ng S. C. (2016). Globalisation of inflammatory bowel disease: perspectives from the evolution of inflammatory bowel disease in the UK and China. Lancet Gastroenterol. Hepatol. 1, 307–316. 10.1016/s2468-1253(16)30077-2 28404201

[B10] Le Berre C. Honap S. Peyrin-Biroulet L. (2023). Ulcerative colitis. Lancet 402, 571–584. 10.1016/s0140-6736(23)00966-2 37573077

[B11] Lee G. R. (2018). The balance of Th17 versus Treg cells in autoimmunity. Int. J. Mol. Sci. 19, 730. 10.3390/ijms19030730 29510522 PMC5877591

[B12] Li H. B. Chen M. Y. Qiu Z. W. Cai Q. Q. Li D. T. Tang H. M. (2018). Efficacy and safety of Kangfuxin liquid combined with aminosalicylic acid for the treatment of ulcerative colitis: a systematic review and meta-analysis. Med. Baltim. 97, e10807. 10.1097/md.0000000000010807 PMC6392804 29794765

[B13] Li N. Lu R. Yu Y. Lu Y. Huang L. Jin J. (2016). Protective effect of *Periplaneta americana* extract in ulcerative colitis rats induced by dinitrochlorobenzene and acetic acid. Pharm. Biol. 54, 2560–2567. 10.3109/13880209.2016.1170862 27309769

[B14] Lv L. Chen Z. Bai W. Hao J. Heng Z. Meng C. (2023). Taurohyodeoxycholic acid alleviates trinitrobenzene sulfonic acid induced ulcerative colitis via regulating Th1/Th2 and Th17/Treg cells balance. Life Sci. 318, 121501. 10.1016/j.lfs.2023.121501 36801213

[B15] Nguyen T. Chen X. Chai J. Li R. Han X. Chen X. (2020). Antipyretic, anti-inflammatory and analgesic activities of *Periplaneta americana* extract and underlying mechanisms. Biomed. Pharmacother. 123, 109753. 10.1016/j.biopha.2019.109753 31865148

[B16] Ordás I. Eckmann L. Talamini M. Baumgart D. C. Sandborn W. J. (2012). Ulcerative colitis. Lancet 380, 1606–1619. 10.1016/s0140-6736(12)60150-0 22914296

[B17] Raine T. Bonovas S. Burisch J. Kucharzik T. Adamina M. Annese V. (2022). ECCO guidelines on therapeutics in ulcerative colitis: medical treatment. J. Crohns Colitis 16, 2–17. 10.1093/ecco-jcc/jjab178 34635919

[B18] Song Q. Gou Q. Xie Y. Zhang Z. Fu C. (2017). *Periplaneta americana* extracts promote skin wound healing via nuclear factor kappa B canonical pathway and extracellular signal-regulated kinase signaling. Evid. Based Complement. Altern. Med. 2017, 5821706. 10.1155/2017/5821706 PMC5460390 28620419

[B19] Tan H. B. Zheng Y. Q. Zhuang Y. P. (2022). IL-17A in diabetic kidney disease: protection or damage. Int. Immunopharmacol. 108, 108707. 10.1016/j.intimp.2022.108707 35344813

[B20] Ungaro R. Mehandru S. Allen P. B. Peyrin-Biroulet L. Colombel J. F. (2017). Ulcerative colitis. Lancet 389, 1756–1770. 10.1016/s0140-6736(16)32126-2 27914657 PMC6487890

[B21] Wang H. Tan H. Zhan W. Song L. Zhang D. Chen X. (2021). Molecular mechanism of Fufang Zhenzhu Tiaozhi capsule in the treatment of type 2 diabetes mellitus with nonalcoholic fatty liver disease based on network pharmacology and validation in minipigs. J. Ethnopharmacol. 274, 114056. 10.1016/j.jep.2021.114056 33771638

[B22] Xiao S. Yan Y. Shao M. Zhou X. Niu Z. Wu Y. (2024). Kuijieling decoction regulates the Treg/Th17 cell balance in ulcerative colitis through the RA/RARα signaling pathway. J. Ethnopharmacol. 318, 116909. 10.1016/j.jep.2023.116909 37451490

[B23] Xie Y. Liang S. Zhang Y. Wu T. Shen Y. Yao S. (2023). Discovery of indole analogues from *Periplaneta americana* extract and their activities on cell proliferation and recovery of ulcerative colitis in mice. Front. Pharmacol. 14, 1282545. 10.3389/fphar.2023.1282545 37927593 PMC10623332

[B24] Yan J. B. Luo M. M. Chen Z. Y. He B. H. (2020). The function and role of the Th17/Treg cell balance in inflammatory bowel disease. J. Immunol. Res. 2020, 8813558. 10.1155/2020/8813558 33381606 PMC7755495

[B25] Yan S. Wang P. Wei H. Jia R. Zhen M. Li Q. (2022). Treatment of ulcerative colitis with Wu-Mei-Wan by inhibiting intestinal inflammatory response and repairing damaged intestinal mucosa. Phytomedicine 105, 154362. 10.1016/j.phymed.2022.154362 35947900

[B26] Yang C. Merlin D. (2024). Unveiling colitis: a journey through the dextran sodium sulfate-induced model. Inflamm. Bowel Dis. 30, 844–853. 10.1093/ibd/izad312 38280217 PMC11063560

[B27] Yang Y. Q. Tan H. B. Zhang X. Y. Zhang Y. Z. Lin Q. Y. Huang M. Y. (2022). The Chinese medicine Fufang Zhenzhu Tiaozhi capsule protects against renal injury and inflammation in mice with diabetic kidney disease. J. Ethnopharmacol. 292, 115165. 10.1016/j.jep.2022.115165 35247475

[B28] Yang Z. Ma J. Li Z. Wang J. Shi Z. (2023). Cellular and molecular mechanisms of Notch signal in pulmonary microvascular endothelial cells after acute lung injury. Braz J. Med. Biol. Res. 56, e12888. 10.1590/1414-431X2023e12888 38126616 PMC10739178

[B29] Yin X. Wei H. Wu S. Wang Z. Liu B. Guo L. (2020). DAPT reverses the Th17/Treg imbalance in experimental autoimmune uveitis *in vitro* via inhibiting Notch signaling pathway. Int. Immunopharmacol. 79, 106107. 10.1016/j.intimp.2019.106107 31863921

[B30] Yu S. Liu C. Li L. Tian T. Wang M. Hu Y. (2015). Inactivation of Notch signaling reverses the Th17/Treg imbalance in cells from patients with immune thrombocytopenia. Lab. Invest 95, 157–167. 10.1038/labinvest.2014.142 25485537

[B31] Zeng C. Liao Q. Hu Y. Shen Y. Geng F. Chen L. (2019). The role of *Periplaneta americana* (Blattodea: Blattidae) in modern versus traditional Chinese medicine. J. Med. Entomol. 56, 1522–1526. 10.1093/jme/tjz081 31265723

