# Supplementary material for: Periplanta americana extract regulates the Th17/Treg cell balance via Notch1 in ulcerative colitis
Source: Front Pharmacol. 2025 Jan 22;15:1534772. doi: 10.3389/fphar.2024.1534772 (PMC11794537; doi:10.3389/fphar.2024.1534772)
Supplement: Supplementary file 1 [file DataSheet1.docx]

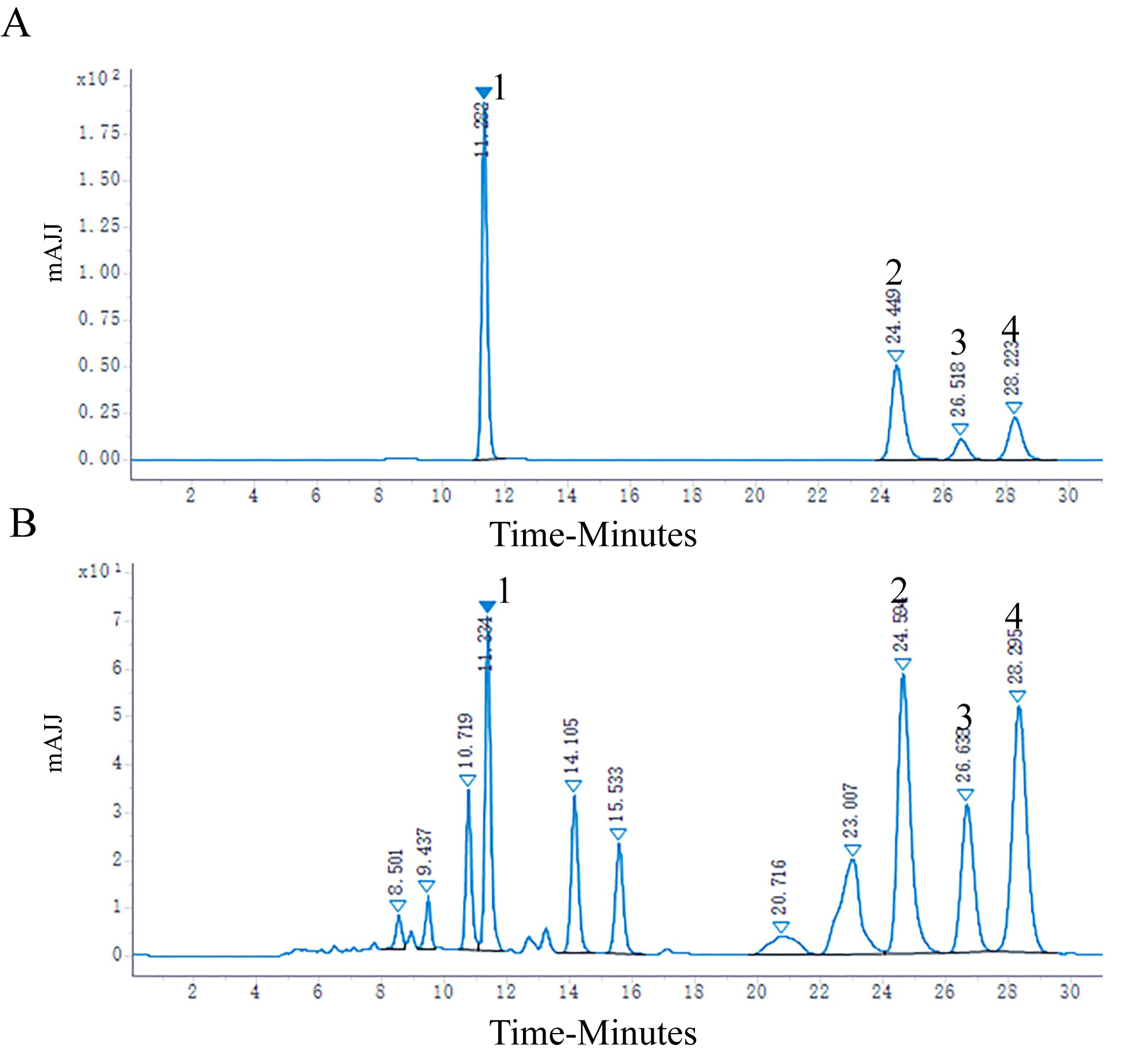
**Supplementary Figure 1.** HPLC chromatogram of mixed reference substance (A) and PAE (B). (1. *uracil*, 2. *hypoxanthine*, 3. *xanthine*, 4. *inosine*)

**Supplementary Table1**

| Score | Weight loss | Stool consistency | Time of occult blood |
| --- | --- | --- | --- |
| 0 | None | Normal | None |
| 1 | 1-5% | ­ | 1-2 mins |
| 2 | 5-10% | Loose stools | 10 s-1 min |
| 3 | 10-20% | ­ | 0-10 s |
| 4 | Over 20 | Watery diarrhea | hematochezia |

**Supplementary Table2**

| Score | Severity of inflammation | Depth of injury | Crypt damage | Percentage of the involved area |
| --- | --- | --- | --- | --- |
| 0 | None | None | Negative | 0% |
| 1 | Slight | Mucosal | Basal 1/3 damaged | 1%-10% |
| 2 | Moderate | Mucosal and submucosal | Basal 2/3 damaged | 10%-25% |
| 3 | Relatively serious | submucosal | Only surface epithelium intact | 25%-50% |
| 4 | Severe | Transmural | Entire crypt and epithelium lost | 50%-100% |

**Supplementary Table 3**

| Compd | Molecular Formula | MW  (g/mol) | Content  (mg/g) | CAS | Molecule ID | Structure |
| --- | --- | --- | --- | --- | --- | --- |
| *uracil* | C_4_H_4_N_2_O_2_ | 112.10 | 1.26 | 66-22-8 | MOL001744 | 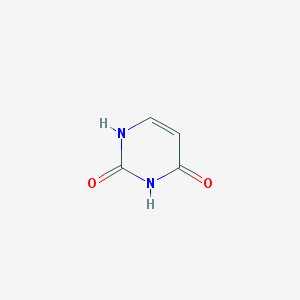 |
| *hypoxanthine* | C_5_H_4_N_4_O | 136.13 | 3.33 | 68-94-0 | MOL001831 | 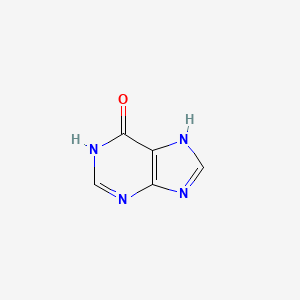 |
| *xanthine* | C_5_H_4_N_4_O_2_ | 152.11 | 3.82 | 69-89-6 | MOL010716 | 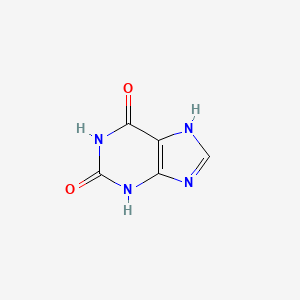 |
| *inosine* | C_10_H_12_N_4_O_5_ | 268.23 | 7.16 | 58-63-9 | MOL006948 | 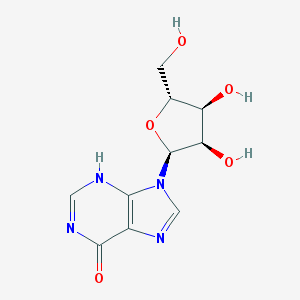 |
